# Crystal structure of bis­(2-methyl-1*H*-imidazol-3-ium) di­hydroxidobis(oxalato-κ^2^ *O* ^1^,*O* ^2^)stannate(IV) monohydrate

**DOI:** 10.1107/S2056989016002061

**Published:** 2016-02-17

**Authors:** Mouhamadou Birame Diop, Libasse Diop, Laurent Plasseraud, Thierry Maris

**Affiliations:** a Laboratoire de Chimie Minérale et Analytique, Département de Chimie, Faculté des Sciences et Techniques, Université Cheikh Anta Diop, Dakar, Senegal; b ICMUB UMR 6302, Université de Bourgogne, Faculté des Sciences, 9 avenue Alain Savary, 21000 Dijon, France; c Département de Chimie, Université de Montréal, 2900 Boulevard Édouard-Montpetit, Montréal, Québec, H3C 3J7, Canada

**Keywords:** crystal structure, organotin(IV) complex, hydrogen bonds

## Abstract

In the structure of the hydrated title salt, (C_4_H_7_N_2_)_2_[Sn(C_2_O_4_)_2_(OH)_2_]·H_2_O, the asymmetric unit comprises one stannate(IV) dianion, two organic cations and one water mol­ecule of crystallization. The [Sn(C_2_O_4_)_2_(OH)_2_]^2−^ dianion consists of an Sn^IV^ atom chelated by two oxalate anions and coordinated by two OH^−^ ligands in a *cis* octa­hedral arrangement. Neighbouring anions are connected through O—H⋯O hydrogen bonds between hydroxide groups and non-coordinating oxalate O atoms into layers expanding parallel to (100). In addition, cations and anions are linked through N—H⋯O hydrogen bonds, and the water mol­ecule bridges two anions with two O—H⋯O hydrogen bonds and is also the acceptor of an N—H⋯O hydrogen bond with one of the cations. Weak C—H⋯O hydrogen bonds are also observed. The intricate hydrogen bonding leads to the formation of a three-dimensional network.

## Abstract 2

The Sn^IV^ atom in the anion of the title compound is six-coordinated by two OH groups and four O atoms from two chelating oxalate ligands. Several N—H⋯O and O—H⋯O hydrogen bonds involving the stannate dianions, the cations and the water mol­ecules result into a three-dimensional network structure.

## Chemical context   

Organotin(IV) compounds are a class of compounds studied for their numerous applications in various fields involving biological activities (Sirajuddin *et al.*, 2014 ▸), biocidal properties (Davies *et al.*, 2008 ▸) or catalysis applications (Meneghetti & Meneghetti, 2015 ▸). Inter­ested in tin(IV) chemistry, our group has so far synthesized and structurally characterized several compounds of this family, see, for example: Sarr *et al.* (2015 ▸); Diop *et al.* (2015 ▸); Gueye *et al.* (2014 ▸). In the course of designing new oxalatostannate(IV) complexes, we report here the result of the reaction between bis­(methyl-2-imidazolium) oxalate and SnCl_2_·2H_2_O that yielded the title compound (C_4_H_7_N_2_)_2_[Sn(C_2_O_4_)_2_(OH)_2_]·H_2_O with tin in oxidation state +IV. A similar oxidation of Sn^II^ to Sn^IV^ has been reported recently (Diop *et al.*, 2015 ▸).
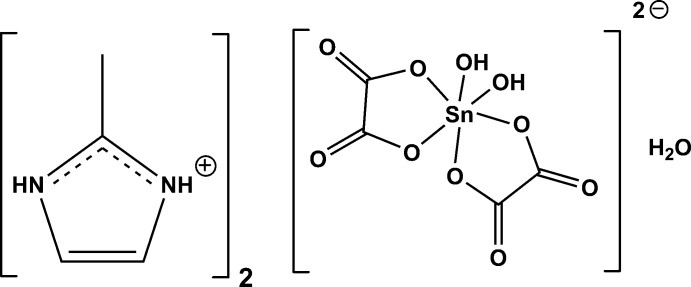


## Structural commentary   

**Figure 1:**
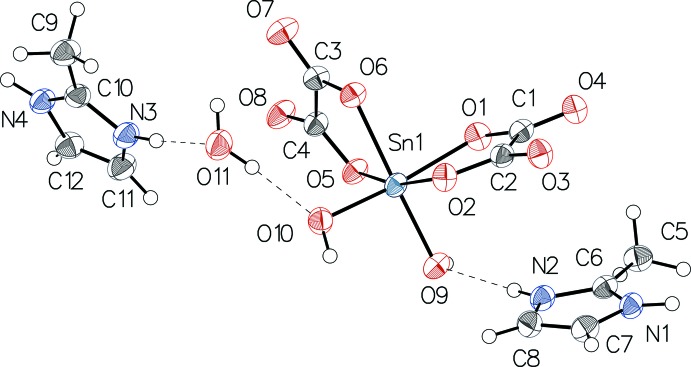
The structure of the mol­ecular components in the title compound, with displacement ellipsoids drawn at the 50% probability level. H atoms are drawn as spheres of arbitrary radius and hydrogen bonds are shown as dashed lines.

The Sn^IV^ atom is chelated by two oxalate anions and is coordinated by two OH groups in a *cis* arrangement, leading to a distorted octa­hedral environment (Fig. 1 ▸). The Sn—O distances involving the oxalate anions [2.103 (2) (O1), 2.077 (2) (O2), 2.074 (2) (O5) and 2.114 (2) Å (O6)] are in the typical range reported for oxalatostannate(IV) anions (Sarr *et al.*, 2015 ▸; Gueye *et al.*, 2014 ▸). The Sn—O distances involving the OH groups [2.001 (2) (O9) and 1.973 (2) Å (O10)] are shorter by *ca* 0.1 Å. The distortion from the ideal octa­hedron is reflected by the *trans* angle O1—Sn—O10 of 169.11 (9)° involving one of the hydroxyl groups and the oxalate O1 atom. Within the oxalate ligands, the distances [C1—O1 1.296 (4), C2—O2 1.300 (4), C3—O6 1.290 (4), C4—O5 1.299 (4) Å] and [C2—O3 1.215 (4), C1—O4 1.223 (4), O7—C3 1.220 (4), O8—C4 1.212 (4) Å] are compatible with single C—O and double C=O bonds, respectively. Bond lengths and angles within the two bis­(2-methyl-1*H*-imidazol-3-ium) cations are in normal ranges.

## Supra­molecular features   

**Figure 2:**
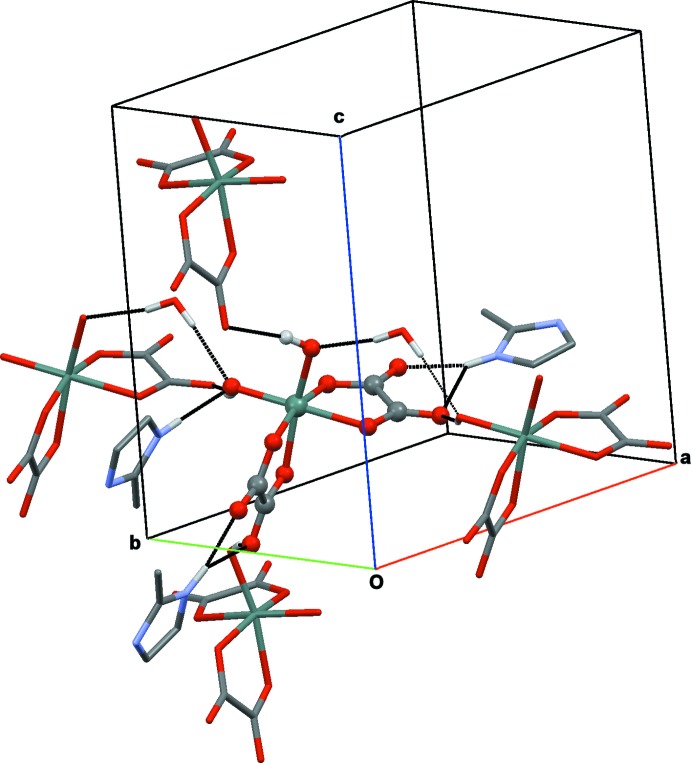
A view of a central stannate dianion (ball-and-stick representation) surrounded by its hydrogen-bonded neighbours (stick representation), *viz* three cations, two water mol­ecules and four other stannate anions. Hydrogen bonds are displayed as black dotted lines and H atoms not involved in hydrogen bonding have been omitted for clarity.

Each stannate dianion [Sn(C_2_O_4_)_2_(OH)_2_]^2−^ is linked to two neighbouring anions through hydrox­yl(OH)⋯O hydrogen bonds involving the non-coordinating oxalate O atoms as acceptor groups. These inter­actions lead to the formation of layers extending parallel to (100). The cations inter­act with the anions *via* N—H⋯O hydrogen bonds (one bifurcated) whereby the non-coordinating oxalate O atoms again are the acceptor groups with the exception of one hydroxyl O atom (O9) as an acceptor (Table 1 ▸). The two hydroxyl groups are also acceptor groups of two (water)OH⋯O inter­actions, giving a total of nine hydrogen-bonding inter­actions per stannate dianion (Fig. 2 ▸). In addition to the dominant classical O—H⋯O and N—H⋯O inter­actions, weak C—H⋯O hydrogen bonds are also present in the structure (Table 1 ▸).

## Synthesis and crystallization   

The title compound was obtained by reacting in methanol in a 2:1 ratio SnCl_2_·2H_2_O with bis­(methyl-2-imidazolium) oxalate. The latter was previously prepared in aqueous solution by mixing in a 2:1 ratio methyl-2-imidazole with oxalic acid and allowing the water to evaporate at 333 K. Slow solvent evaporation at room temperature afforded colourless crystals suitable for X-ray diffraction analysis.

## Refinement   

Crystal data, data collection and structure refinement details are summarized in Table 2 ▸. The coordinates of H atoms of the water mol­ecules and hy­droxy groups were obtained from a difference map and were refined using SADI and DFIX restraints (Sheldrick, 2015*b* ▸). All other H atoms were positioned geometrically (C—H = 0.95, 0.98 Å, N—H = 0.88 Å) and refined as riding with *U*_iso_(H) = *xU*_eq_(C, N) with *x* = 1.5 for methyl and *x* = 1.2 for other H atoms.

## Supplementary Material

Crystal structure: contains datablock(s) I, global. DOI: 10.1107/S2056989016002061/wm5268sup1.cif

Structure factors: contains datablock(s) I. DOI: 10.1107/S2056989016002061/wm5268Isup2.hkl

CCDC reference: 1451548

Additional supporting information:  crystallographic information; 3D view; checkCIF report

## Figures and Tables

**Table 1:** Hydrogen-bond geometry (Å, °)

*D*—H⋯*A*	*D*—H	H⋯*A*	*D*⋯*A*	*D*—H⋯*A*
O9—H9⋯O7^i^	0.86	2.00	2.835 (3)	163
O10—H10⋯O4^ii^	0.87	2.06	2.909 (3)	167
O11—H11*A*⋯O10	0.83 (2)	1.95 (2)	2.766 (4)	169 (5)
O11—H11*B*⋯O9^iii^	0.83 (2)	2.10 (2)	2.914 (4)	170 (7)
N1—H1⋯O3^iv^	0.88	1.97	2.793 (4)	156
N1—H1⋯O4^iv^	0.88	2.50	3.131 (3)	129
N2—H2⋯O9	0.88	1.90	2.742 (3)	160
N3—H3⋯O11	0.88	1.84	2.713 (4)	175
N4—H4⋯O8^v^	0.88	1.94	2.787 (4)	161
C5—H5*A*⋯O4	0.98	2.55	3.460 (4)	155
C7—H7⋯O2^vi^	0.95	2.39	3.327 (4)	169
C8—H8⋯O4^ii^	0.95	2.58	3.444 (4)	152
C12—H12⋯O5^vii^	0.95	2.33	3.232 (4)	159

Symmetry codes: (i) 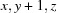; (ii) 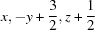; (iii) 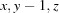; (iv) 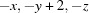; (v) 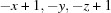; (vi) 
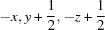
; (vii) 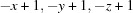.

**Table 2:** Experimental details

Crystal data	
Chemical formula	(C_4_H_7_N_2_)_2_[Sn(C_2_O_4_)_2_(H_2_O)_2_]·H_2_O
*M*_r_	512.99
Crystal system, space group	Monoclinic, *P*2_1_/*c*
Temperature (K)	110
*a*, *b*, *c* (Å)	20.1391 (13), 7.0651 (5), 13.4942 (9)
β (°)	106.582 (2)
*V* (Å^3^)	1840.2 (2)
*Z*	4
Radiation type	Ga *K*α, λ = 1.34139 Å
μ (mm^−1^)	7.83
Crystal size (mm)	0.19 × 0.11 × 0.09

Data collection	
Diffractometer	Bruker Venture Metaljet
Absorption correction	Multi-scan (*SADABS*; Krause *et al.*, 2015 ▸)
*T*_min_, *T*_max_	0.509, 0.752
No. of measured, independent and observed [*I* > 2σ(*I*)] reflections	42907, 4235, 4110
*R*_int_	0.058
(sin θ/λ)_max_ (Å^−1^)	0.651

Refinement	
*R*[*F* ^2^ > 2σ(*F* ^2^)], *wR*(*F* ^2^), *S*	0.040, 0.101, 1.07
No. of reflections	4235
No. of parameters	265
No. of restraints	4
H-atom treatment	H atoms treated by a mixture of independent and constrained refinement
Δρ_max_, Δρ_min_ (e Å^−3^)	1.87, −0.81

Computer programs: *APEX2* and *SAINT* (Bruker, 2014 ▸), *SHELXT* (Sheldrick, 2015*a* ▸), *SHELXL2014* (Sheldrick, 2015*b* ▸), *OLEX2* (Dolomanov *et al.*, 2009 ▸), *Mercury* (Macrae *et al.*, 2008 ▸) and *publCIF* (Westrip, 2010 ▸).

## supplementary crystallographic information

### Crystal data

**Table:**

(C_4_H_7_N_2_)_2_[Sn(C_2_O_4_)_2_(H_2_O)_2_]·H_2_O	*F*(000) = 1024
*M**_r_* = 512.99	*D*_x_ = 1.852 Mg m^−^^3^
Monoclinic, *P*2_1_/*c*	Ga *K*α radiation, λ = 1.34139 Å
*a* = 20.1391 (13) Å	Cell parameters from 9589 reflections
*b* = 7.0651 (5) Å	θ = 4.2–60.8°
*c* = 13.4942 (9) Å	µ = 7.83 mm^−^^1^
β = 106.582 (2)°	*T* = 110 K
*V* = 1840.2 (2) Å^3^	Block, clear light colourless
*Z* = 4	0.19 × 0.11 × 0.09 mm

### Data collection

**Table:**

Bruker Venture Metaljet diffractometer	4235 independent reflections
Radiation source: Metal Jet, Gallium Liquid Metal Jet Source	4110 reflections with *I* > 2σ(*I*)
Helios MX Mirror Optics monochromator	*R*_int_ = 0.058
Detector resolution: 10.24 pixels mm^-1^	θ_max_ = 60.9°, θ_min_ = 2.0°
ω and φ scans	*h* = −26→26
Absorption correction: multi-scan (*SADABS*; Krause *et al.*, 2015)	*k* = −8→9
*T*_min_ = 0.509, *T*_max_ = 0.752	*l* = −17→17
42907 measured reflections	

### Refinement

**Table:**

Refinement on *F*^2^	Primary atom site location: structure-invariant direct methods
Least-squares matrix: full	Hydrogen site location: mixed
*R*[*F*^2^ > 2σ(*F*^2^)] = 0.040	H atoms treated by a mixture of independent and constrained refinement
*wR*(*F*^2^) = 0.101	*w* = 1/[σ^2^(*F*_o_^2^) + (0.0612*P*)^2^ + 2.7674*P*] where *P* = (*F*_o_^2^ + 2*F*_c_^2^)/3
*S* = 1.07	(Δ/σ)_max_ = 0.001
4235 reflections	Δρ_max_ = 1.87 e Å^−^^3^
265 parameters	Δρ_min_ = −0.81 e Å^−^^3^
4 restraints	

### Special details

**Table:**

Experimental. X-ray crystallographic data for I were collected from a single-crystal sample, which was mounted on a loop fiber. Data were collected using a Bruker Venture diffractometer equipped with a Photon 100 CMOS Detector, a Helios MX optics and a Kappa goniometer. The crystal-to-detector distance was 4.0 cm, and the data collection was carried out in 1024 *x* 1024 pixel mode.
Geometry. All e.s.d.'s (except the e.s.d. in the dihedral angle between two l.s. planes) are estimated using the full covariance matrix. The cell e.s.d.'s are taken into account individually in the estimation of e.s.d.'s in distances, angles and torsion angles; correlations between e.s.d.'s in cell parameters are only used when they are defined by crystal symmetry. An approximate (isotropic) treatment of cell e.s.d.'s is used for estimating e.s.d.'s involving l.s. planes.

### Fractional atomic coordinates and isotropic or equivalent isotropic displacement parameters (Å^2^)

**Table:**

	*x*	*y*	*z*	*U*_iso_*/*U*_eq_	
Sn1	0.24060 (2)	0.62119 (3)	0.27535 (2)	0.02481 (10)	
O1	0.22492 (11)	0.6762 (3)	0.11720 (17)	0.0312 (4)	
O2	0.13386 (12)	0.5897 (3)	0.21916 (18)	0.0293 (5)	
O3	0.04507 (12)	0.6473 (3)	0.07971 (18)	0.0330 (5)	
O4	0.13911 (12)	0.7325 (3)	−0.02786 (17)	0.0347 (5)	
O5	0.34706 (12)	0.6212 (3)	0.29967 (19)	0.0278 (5)	
O6	0.25904 (10)	0.3368 (3)	0.24215 (18)	0.0280 (4)	
O7	0.34600 (13)	0.1302 (3)	0.2613 (2)	0.0368 (6)	
O8	0.43752 (11)	0.4264 (3)	0.3297 (2)	0.0347 (5)	
O9	0.24047 (12)	0.9006 (3)	0.2994 (2)	0.0306 (5)	
H9	0.2722	0.9540	0.2777	0.046*	
O10	0.24113 (12)	0.5339 (3)	0.41448 (17)	0.0321 (5)	
H10	0.2163	0.6085	0.4403	0.048*	
C1	0.16053 (15)	0.6868 (4)	0.0632 (2)	0.0274 (6)	
C2	0.10678 (17)	0.6380 (4)	0.1231 (2)	0.0273 (6)	
C3	0.32352 (15)	0.2896 (4)	0.2648 (2)	0.0280 (6)	
C4	0.37567 (15)	0.4559 (4)	0.3017 (2)	0.0275 (6)	
N1	0.01942 (13)	1.1771 (4)	0.1086 (2)	0.0289 (5)	
H1	−0.0115	1.2288	0.0559	0.043*	
N2	0.11571 (14)	1.0703 (4)	0.2068 (2)	0.0299 (5)	
H2	0.1597	1.0387	0.2303	0.045*	
C5	0.12035 (18)	1.2002 (5)	0.0351 (3)	0.0343 (7)	
H5A	0.1243	1.0864	−0.0043	0.051*	
H5B	0.1667	1.2509	0.0683	0.051*	
H5C	0.0928	1.2956	−0.0116	0.051*	
C6	0.08607 (16)	1.1515 (4)	0.1151 (2)	0.0271 (6)	
C7	0.00616 (19)	1.1103 (4)	0.1971 (3)	0.0332 (7)	
H7	−0.0372	1.1110	0.2118	0.040*	
C8	0.06634 (17)	1.0440 (5)	0.2585 (2)	0.0334 (6)	
H8	0.0736	0.9893	0.3250	0.040*	
N3	0.40483 (15)	0.0861 (4)	0.5690 (2)	0.0342 (6)	
H3	0.3611	0.1160	0.5404	0.051*	
N4	0.49720 (14)	−0.0750 (4)	0.6344 (2)	0.0306 (5)	
H4	0.5252	−0.1712	0.6567	0.046*	
C9	0.38714 (19)	−0.2637 (5)	0.5737 (3)	0.0390 (7)	
H9A	0.4017	−0.3457	0.6346	0.059*	
H9B	0.3941	−0.3298	0.5136	0.059*	
H9C	0.3380	−0.2320	0.5604	0.059*	
C10	0.42895 (17)	−0.0874 (5)	0.5925 (2)	0.0298 (6)	
C11	0.45913 (19)	0.2124 (5)	0.5965 (3)	0.0380 (7)	
H11	0.4562	0.3460	0.5881	0.046*	
C12	0.5171 (2)	0.1116 (4)	0.6374 (3)	0.0347 (7)	
H12	0.5628	0.1599	0.6633	0.042*	
O11	0.26942 (14)	0.1609 (4)	0.4740 (2)	0.0406 (6)	
H11A	0.258 (3)	0.267 (3)	0.449 (4)	0.074 (18)*	
H11B	0.259 (4)	0.079 (6)	0.428 (4)	0.10 (2)*	

### Atomic displacement parameters (Å^2^)

**Table:**

	*U*^11^	*U*^22^	*U*^33^	*U*^12^	*U*^13^	*U*^23^
Sn1	0.01935 (13)	0.02397 (14)	0.02993 (14)	0.00241 (6)	0.00517 (9)	0.00129 (6)
O1	0.0245 (10)	0.0368 (11)	0.0333 (11)	−0.0011 (9)	0.0097 (8)	0.0009 (9)
O2	0.0236 (10)	0.0338 (11)	0.0311 (11)	0.0006 (9)	0.0088 (9)	0.0052 (9)
O3	0.0233 (11)	0.0407 (12)	0.0327 (11)	−0.0004 (9)	0.0043 (9)	0.0021 (9)
O4	0.0353 (12)	0.0380 (12)	0.0295 (11)	0.0011 (9)	0.0073 (9)	0.0035 (9)
O5	0.0205 (10)	0.0245 (11)	0.0389 (12)	0.0019 (7)	0.0092 (9)	0.0008 (8)
O6	0.0216 (10)	0.0263 (10)	0.0357 (11)	−0.0001 (8)	0.0075 (8)	−0.0016 (9)
O7	0.0286 (12)	0.0265 (12)	0.0561 (16)	0.0006 (8)	0.0136 (11)	−0.0043 (9)
O8	0.0218 (10)	0.0284 (10)	0.0524 (14)	0.0023 (9)	0.0080 (10)	0.0003 (10)
O9	0.0266 (12)	0.0241 (10)	0.0408 (12)	0.0015 (8)	0.0090 (9)	0.0010 (9)
O10	0.0329 (11)	0.0344 (12)	0.0297 (10)	0.0044 (9)	0.0100 (9)	0.0037 (9)
C1	0.0257 (14)	0.0237 (14)	0.0321 (14)	0.0011 (11)	0.0070 (11)	−0.0011 (11)
C2	0.0263 (15)	0.0241 (14)	0.0295 (14)	−0.0003 (10)	0.0046 (12)	−0.0011 (10)
C3	0.0250 (14)	0.0259 (14)	0.0335 (14)	−0.0011 (11)	0.0089 (11)	−0.0020 (11)
C4	0.0253 (14)	0.0256 (14)	0.0311 (14)	0.0012 (11)	0.0071 (11)	0.0005 (11)
N1	0.0238 (12)	0.0302 (13)	0.0311 (12)	0.0037 (10)	0.0055 (10)	0.0016 (10)
N2	0.0241 (12)	0.0287 (12)	0.0342 (13)	0.0026 (10)	0.0039 (10)	−0.0011 (11)
C5	0.0340 (16)	0.0342 (17)	0.0375 (16)	−0.0007 (13)	0.0146 (13)	−0.0016 (13)
C6	0.0242 (14)	0.0246 (13)	0.0324 (15)	0.0008 (11)	0.0077 (12)	−0.0019 (11)
C7	0.0300 (17)	0.0335 (17)	0.0377 (17)	0.0005 (12)	0.0122 (14)	−0.0001 (12)
C8	0.0378 (17)	0.0298 (16)	0.0319 (15)	−0.0003 (13)	0.0086 (13)	0.0019 (12)
N3	0.0305 (14)	0.0313 (13)	0.0377 (14)	0.0056 (11)	0.0048 (11)	−0.0020 (11)
N4	0.0262 (13)	0.0274 (12)	0.0360 (14)	0.0032 (11)	0.0052 (11)	−0.0020 (11)
C9	0.0348 (17)	0.0329 (17)	0.0463 (18)	−0.0007 (14)	0.0067 (14)	−0.0071 (14)
C10	0.0266 (15)	0.0309 (14)	0.0313 (15)	0.0035 (12)	0.0071 (12)	−0.0041 (12)
C11	0.0416 (18)	0.0270 (15)	0.0443 (18)	0.0000 (13)	0.0106 (15)	−0.0007 (13)
C12	0.0328 (17)	0.0324 (17)	0.0384 (17)	−0.0040 (12)	0.0092 (14)	−0.0029 (12)
O11	0.0375 (13)	0.0423 (13)	0.0409 (13)	0.0100 (11)	0.0095 (11)	0.0049 (12)

### Geometric parameters (Å, º)

**Table:**

Sn1—O1	2.103 (2)	N2—C8	1.380 (4)
Sn1—O2	2.077 (2)	C5—H5A	0.9800
Sn1—O5	2.074 (2)	C5—H5B	0.9800
Sn1—O6	2.114 (2)	C5—H5C	0.9800
Sn1—O9	2.001 (2)	C5—C6	1.478 (4)
Sn1—O10	1.973 (2)	C7—H7	0.9500
O1—C1	1.296 (4)	C7—C8	1.342 (5)
O2—C2	1.300 (4)	C8—H8	0.9500
O3—C2	1.215 (4)	N3—H3	0.8800
O4—C1	1.223 (4)	N3—C10	1.324 (4)
O5—C4	1.299 (4)	N3—C11	1.378 (5)
O6—C3	1.290 (4)	N4—H4	0.8800
O7—C3	1.220 (4)	N4—C10	1.332 (4)
O8—C4	1.212 (4)	N4—C12	1.375 (4)
O9—H9	0.8616	C9—H9A	0.9800
O10—H10	0.8653	C9—H9B	0.9800
C1—C2	1.563 (4)	C9—H9C	0.9800
C3—C4	1.560 (4)	C9—C10	1.484 (5)
N1—H1	0.8800	C11—H11	0.9500
N1—C6	1.332 (4)	C11—C12	1.344 (5)
N1—C7	1.379 (4)	C12—H12	0.9500
N2—H2	0.8800	O11—H11A	0.831 (15)
N2—C6	1.339 (4)	O11—H11B	0.827 (16)
			
O1—Sn1—O6	86.86 (9)	C8—N2—H2	125.4
O2—Sn1—O1	79.01 (9)	H5A—C5—H5B	109.5
O2—Sn1—O6	92.69 (8)	H5A—C5—H5C	109.5
O5—Sn1—O1	90.55 (9)	H5B—C5—H5C	109.5
O5—Sn1—O2	166.65 (9)	C6—C5—H5A	109.5
O5—Sn1—O6	78.32 (8)	C6—C5—H5B	109.5
O9—Sn1—O1	88.58 (10)	C6—C5—H5C	109.5
O9—Sn1—O2	96.65 (9)	N1—C6—N2	107.1 (3)
O9—Sn1—O5	91.33 (8)	N1—C6—C5	126.3 (3)
O9—Sn1—O6	168.64 (9)	N2—C6—C5	126.6 (3)
O10—Sn1—O1	169.11 (9)	N1—C7—H7	126.6
O10—Sn1—O2	92.17 (9)	C8—C7—N1	106.8 (3)
O10—Sn1—O5	97.17 (9)	C8—C7—H7	126.6
O10—Sn1—O6	87.19 (9)	N2—C8—H8	126.4
O10—Sn1—O9	98.87 (10)	C7—C8—N2	107.2 (3)
C1—O1—Sn1	114.71 (19)	C7—C8—H8	126.4
C2—O2—Sn1	115.6 (2)	C10—N3—H3	125.5
C4—O5—Sn1	115.80 (18)	C10—N3—C11	109.1 (3)
C3—O6—Sn1	114.93 (19)	C11—N3—H3	125.5
Sn1—O9—H9	110.0	C10—N4—H4	125.3
Sn1—O10—H10	110.2	C10—N4—C12	109.4 (3)
O1—C1—C2	115.2 (3)	C12—N4—H4	125.3
O4—C1—O1	126.1 (3)	H9A—C9—H9B	109.5
O4—C1—C2	118.7 (3)	H9A—C9—H9C	109.5
O2—C2—C1	114.7 (3)	H9B—C9—H9C	109.5
O3—C2—O2	125.1 (3)	C10—C9—H9A	109.5
O3—C2—C1	120.2 (3)	C10—C9—H9B	109.5
O6—C3—C4	114.9 (3)	C10—C9—H9C	109.5
O7—C3—O6	126.1 (3)	N3—C10—N4	107.7 (3)
O7—C3—C4	119.0 (3)	N3—C10—C9	125.8 (3)
O5—C4—C3	114.6 (2)	N4—C10—C9	126.5 (3)
O8—C4—O5	124.8 (3)	N3—C11—H11	126.4
O8—C4—C3	120.6 (3)	C12—C11—N3	107.3 (3)
C6—N1—H1	125.2	C12—C11—H11	126.4
C6—N1—C7	109.7 (3)	N4—C12—H12	126.7
C7—N1—H1	125.2	C11—C12—N4	106.6 (3)
C6—N2—H2	125.4	C11—C12—H12	126.7
C6—N2—C8	109.2 (3)	H11A—O11—H11B	110 (3)
			
Sn1—O1—C1—O4	173.7 (3)	O7—C3—C4—O8	−2.5 (5)
Sn1—O1—C1—C2	−5.5 (3)	N1—C7—C8—N2	−0.2 (4)
Sn1—O2—C2—O3	−172.4 (2)	C6—N1—C7—C8	0.4 (4)
Sn1—O2—C2—C1	7.4 (3)	C6—N2—C8—C7	0.0 (4)
Sn1—O5—C4—O8	−168.6 (3)	C7—N1—C6—N2	−0.4 (4)
Sn1—O5—C4—C3	11.0 (3)	C7—N1—C6—C5	178.5 (3)
Sn1—O6—C3—O7	172.8 (3)	C8—N2—C6—N1	0.2 (4)
Sn1—O6—C3—C4	−6.1 (3)	C8—N2—C6—C5	−178.7 (3)
O1—C1—C2—O2	−1.2 (4)	N3—C11—C12—N4	−0.1 (4)
O1—C1—C2—O3	178.6 (3)	C10—N3—C11—C12	0.0 (4)
O4—C1—C2—O2	179.6 (3)	C10—N4—C12—C11	0.2 (4)
O4—C1—C2—O3	−0.7 (4)	C11—N3—C10—N4	0.1 (4)
O6—C3—C4—O5	−3.2 (4)	C11—N3—C10—C9	−179.0 (3)
O6—C3—C4—O8	176.5 (3)	C12—N4—C10—N3	−0.2 (4)
O7—C3—C4—O5	177.8 (3)	C12—N4—C10—C9	179.0 (3)

### Hydrogen-bond geometry (Å, º)

**Table:**

*D*—H···*A*	*D*—H	H···*A*	*D*···*A*	*D*—H···*A*
O9—H9···O7^i^	0.86	2.00	2.835 (3)	163
O10—H10···O4^ii^	0.87	2.06	2.909 (3)	167
O11—H11*A*···O10	0.83 (2)	1.95 (2)	2.766 (4)	169 (5)
O11—H11*B*···O9^iii^	0.83 (2)	2.10 (2)	2.914 (4)	170 (7)
N1—H1···O3^iv^	0.88	1.97	2.793 (4)	156
N1—H1···O4^iv^	0.88	2.50	3.131 (3)	129
N2—H2···O9	0.88	1.90	2.742 (3)	160
N3—H3···O11	0.88	1.84	2.713 (4)	175
N4—H4···O8^v^	0.88	1.94	2.787 (4)	161
C5—H5*A*···O4	0.98	2.55	3.460 (4)	155
C7—H7···O2^vi^	0.95	2.39	3.327 (4)	169
C8—H8···O4^ii^	0.95	2.58	3.444 (4)	152
C12—H12···O5^vii^	0.95	2.33	3.232 (4)	159

Symmetry codes: (i) *x*, *y*+1, *z*; (ii) *x*, −*y*+3/2, *z*+1/2; (iii) *x*, *y*−1, *z*; (iv) −*x*, −*y*+2, −*z*; (v) −*x*+1, −*y*, −*z*+1; (vi) −*x*, *y*+1/2, −*z*+1/2; (vii) −*x*+1, −*y*+1, −*z*+1.
